# MRI image analysis methods and applications: an algorithmic perspective using brain tumors as an exemplar

**DOI:** 10.1093/noajnl/vdaa049

**Published:** 2020-04-14

**Authors:** Vachan Vadmal, Grant Junno, Chaitra Badve, William Huang, Kristin A Waite, Jill S Barnholtz-Sloan

**Affiliations:** 1 Department of Population Health and Quantitative Sciences, Case Western Reserve University School of Medicine, Cleveland, Ohio; 2 Cleveland Center for Health Outcomes Research (CCHOR), Cleveland, Ohio; 3 Department of Radiology, University Hospitals Health System (UHHS), Cleveland, Ohio; 4 Research Health Analytics and Informatics, UHHS, Cleveland, Ohio; 5 Case Comprehensive Cancer Center, Cleveland, Ohio; 6 Cleveland Institute for Computational Biology, Cleveland, Ohio

## Abstract

The use of magnetic resonance imaging (MRI) in healthcare and the emergence of radiology as a practice are both relatively new compared with the classical specialties in medicine. Having its naissance in the 1970s and later adoption in the 1980s, the use of MRI has grown exponentially, consequently engendering exciting new areas of research. One such development is the use of computational techniques to analyze MRI images much like the way a radiologist would. With the advent of affordable, powerful computing hardware and parallel developments in computer vision, MRI image analysis has also witnessed unprecedented growth. Due to the interdisciplinary and complex nature of this subfield, it is important to survey the current landscape and examine the current approaches for analysis and trend trends moving forward.

**Key Points**

- MRI imaging, analytics, imaging informatics, deep learning.

The past decade has seen a remarkable change in the availability of powerful, inexpensive computer hardware that has been a major driving force for the progression of machine vision in medical research. This has resulted in advances in digital MRI imaging analysis that ranges from simple tumor identification to the assessment of tumor response and treatments in clinical oncology.^1^ Due to the interdisciplinary nature of the field, principles from physics, computer science, and computer graphics are used to address medical imaging informatics problems. With the existence of vast amounts of imaging data procured during standard clinical practice, a primary focus among investigators has been to use image analysis to augment current standards of tumor detection and to gain new insights about the nature of diseases. The stages in a typical workflow are image acquisition, preprocessing, segmentation, and feature extraction. These key terms that define a typical workflow were queried to find current literature in repositories such as Elsevier, IEEE Xplore, Radiology, PubMed, and Google Scholar. This review discusses past and current methods employed in each of these stages as well as the rising popularity of artificial intelligence (AI)-based approaches, using brain tumors as an exemplar. A glossary of key terms is provided in the supplementary materials for ease of reference as these topics are presented.

## Preprocessing

The first step in any data-driven study is to preprocess the raw images. Preprocessing removes noise by ensuring there is a degree of parity among all the images that in turn make the following segmentation and feature extraction steps more effective.^2^ This involves performing operations to remove artifacts, modify image resolution, and address contrast differences that arise from different acquisition hardware and parameters. One common source of noise is bias fields, which are caused by low-frequency signals emitted from the MRI machine combined with patient anatomy that ultimately leads to inhomogeneities in the magnetic field.^3^ The resulting images, therefore, have variations in intensity for the same tissue when each tissue should correspond to a specific intensity level.^4,5^ Another source of noise arises from temporal data. During the course of treatment, patients often have a series of pre- and post-images. These imaging series are valuable for analytics, but is almost impossible for the patient to be in the same exact position for the pre- and post-scans. This can make it difficult to discern the status of the tumor not only for imaging software, but also for radiologists. Thus, images taken over a timeframe must be aligned in a process known as image registration.

To address the contrast differences in studies where images are taken from multiple sources and machines, images undergo normalization of color or grayscale values.^6^ Normalization is almost universal in controlled imaging studies and is necessary when employing machine-learning techniques. Normalization effectively defines a new range of color values relative to other images in the data set. Before normalization, it may be necessary to remove noise existing on scans of any modality, including the signal from the patient’s skull for patients with brain tumors. Skull stripping is employed to reduce noise from the scans and increase the signal intensities.

### MR Bias Correction

Despite the use of higher field strength MRI scanners, inhomogeneities in the magnetic field coupled with general anatomical noise from tissue attenuation will result in minute, visibly undetectable intensity variations in the resulting images.^5,7^ Because these nonuniformities can skew results of segmentation and statistical features detection, they need to be corrected before proceeding with the rest of the analytical pipeline.^5^ The 2 main methodologies for reducing bias field are prospective and retrospective methods.^3^ Prospective approaches attempt to reduce the bias field by altering the image capture sequence on the MRI hardware side. Retrospective approaches apply post processing strategies on the already captured image. Retrospective methods can be classified into 4 main subcategories: filtering, surface fitting, segmentation, and histogram.

#### Filtering Methods

Filtering-based methods are perhaps the oldest, easiest, and least computationally demanding of the 4 categories. Filtering removes aspects that meet or do not meet a specified threshold. For MR images, the noise that is removed are artifacts corresponding to low frequencies. However, because the filtering is rather crude, there is a high probability of removing valid signals when using low-pass filtering techniques and the chance of creating new artifacts called edge effects. Research has been conducted to mitigate edge effects, but the overall result still shows bias field.^3^ This is important when analyzing brain tumor images as it is crucial to properly identify the structural differences that change as the disease progresses such as the necrotic area and the tumor.

The 2 main classical filtering methods still used today are homomorphic filtering and homomorphic unsharp masking. Here, the image is first log transformed followed by a transformation into the frequency domain. Then the bias field is removed via a low-pass filter with the corrected image being the difference between the original image and the bias field. This bias field image is often called the background image.^4^ Homomorphic unsharp masking performs the same operations without log transforming the image.

#### Surface Fitting

The surface fitting approach is parametric in that it attempts to extract the background image by representing the image as a parametric surface and fitting a 2D image to it.^3,7^ The 2 main categories of surface fitting methods are intensity and gradient based. Intensity-based methods operate under the precondition that there is no significant intensity variation for a single tissue type. Similarly, gradient-based methods operate with the assumption that there is an even dispersion of bias field and are corrected by estimating the variation in intensity gradients.^3^

#### Segmentation

Because accurate segmentation of regions of interest (ROI) is the goal of bias correction, the 2 steps can be combined. The 2 main segmentation-based approaches are both iterative algorithms: expectation maximum (EM) and fuzzy c-means. The EM algorithm is a machine learning-based approach used to iteratively converge a parametric model’s parameters based on the maximum likelihood probability. The EM approach can use different criteria to estimate the model’s parameters. The fuzzy c-means method also iteratively segments by minimizing a cost function as it steps through a vector of the image’s pixel intensities.^4^ EM-based approaches have fallen out of in favor of fuzzy c-means.

#### Histogram

**Figure 1.**
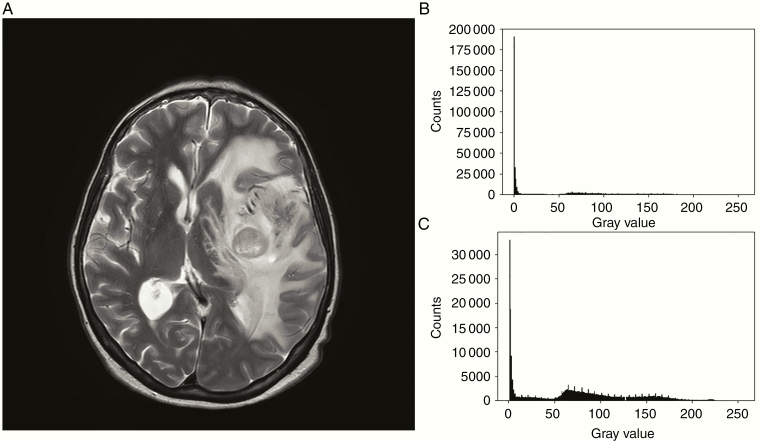
(a) An axial slice near the middle of the brain and its associated histograms. (b) A histogram of all gray-level values (0–255). (c) A histogram of all gray-level values but 0 (1–255).

A histogram is a list that runs the length of the number of intensity values and counts the frequency of each pixel intensity for a given image. An example of a histogram showing the 8-bit pixel value distribution of a slice can be seen in Figure 1b and 1c. Approaches that use intensity distributions are popular and a standard way many research studies correct bias in MR images.^3^ The nonparametric nonuniform normalization method (N3) has, since its inception in 1998, been shown to produce the best bias correction. Since then, the N3 method has been upgraded, and the current standard for bias correction is the N4 method. A popular software that contains the N4 bias correction can be found in the Nipype Python package. Chang and coworkers used the N4 bias correction in deep learning based study utilizing TensorFlow to predict isocitrate dehydrogenase status in low- and high-grade gliomas.^8^ Although there are several approaches to address bias correction, the area still remains one of the active researches.

### Image Registration

Image registration is the process by which 2 images are spatially aligned using a combination of geometric transformations governed by an optimizer. An image can be geometrically represented and transformed in multiple ways, each with its own pros and cons. It is crucial that key biological landmarks are in the same location for an accurate comparison and analysis. For example, studies may have multitemporal (occurring over a period of time) and/or multimodal (having different contrasts) patient MR imaging data. Due to the breadth of the different kinds of problems that exist when registering images, no one method works for all cases.^9,10^ In cases that involve brain tumors, especially well-defined glioblastoma multiforme, image registration is crucial as the extraction of accurate morphological features depends on correct alignment of the tumor region.

Registration can be divided into 4 main components: feature space, search space, search strategy, and the similarity. Each provides vital information to determine which registration technique to use.^10^ Feature space refers to the area of interest to be used as the basis for registration, for example, edges, outlines, tumors. Search space refers to how the image will be transformed to align with the source. Search strategy follows up by determining what transformation to choose based on previous transformation results. The similarity is a comparison metric between the source and target images that are being aligned. This forms the basis of how to frame the registration problem. Recently, advances in image registration research has made this less experimental and more applicable.

In practice, the de facto standard for research-based MR image registration and segmentation utilizes the software suite, Insight ToolKit (ITK).^9^ ITK (version 5.0) consists of a robust set of algorithms and a structured used in many medical imaging-based software such as 3D Slicer and ITK-Snap. In addition to ITK, the FMRIB Software Library (^11^FSL) also offers a set of robust image registration frameworks; FMRIB’s Linear Image Registration Tool and its nonlinear counterpart, FMRIB’s Nonlinear Image Registration Tool. Both ITK and FSL are highly regarded for registration. Links to the mentioned software can be found in the Supplementary Materials.

#### Traditional Registration

##### Principle Axis Transformation

Principal axes transformation, first reported in 1990s, is a classical way of registering images based off the rigid body rotation concept in Newtonian dynamics.^11^ Using brain tumors as the exemplar, we start with the brain. The rigid body is the overall shape of the brain. The brain is treated as a body of mass (ellipse), exhibiting the properties of a mass body such as a center of mass. In this algorithm, the center of mass, or centroid, of the head is calculated. It is important to note that the centroid is computed from the bounding surface of the brain and not the actual dimensions of the image. This is computed by finding the mean intensity level for the *x* and *y* axes. Calculated by^11^:

$$\begin{array}{l} \hat{x}=\frac{\Sigma x*I(x,y)}{I(x,y)},\hat{y}=\frac{\Sigma y*I(x,y)}{I(x,y)} \end{array}$$

where *I* refers to the intensity of the pixel at coordinate (*x*, *y*). The moment of inertia matrix of the rigid body is also calculated. This is a standard property of the rigid body that describes the rotational moment from the center of mass. The eigenvector column vectors are then calculated from the inertia matrix, that is then used to find the axes of the ellipse of the head. This is done for both target and source images. The maximum eigenvector is used to calculate the angle with the horizontal axes, which is then compared against source and target images. The difference in angle between source and target is used to dictate how much to align the source to the target.^11^ Advantages of this algorithm are as follows: (1) it is easier to register images of different contrasts (modalities), for example PD to T2, and (2) it is a completely unsupervised process.

#### Finite Fourier Transform

Another unsupervised, rigid-body-based method utilizes a comparison of the source and target images via the frequency domain through Fourier transformations.^12^ The basis of this algorithm is that given 2 images, the source *s*_0_ (*x*, *y*) and the target or translated image *s*_1_ (*x*, *y*), where the target *s*_1_ is assumed to be rotated by an angle *θ* and translated by pixel distances (Δ*x*, Δ*y*).^12^ Thus, the problem now is to find the translation distances (Δ*x*, Δ*y*) and *θ*, which is accomplished by Fourier transforming *s*_0_ (*x*, *y*) and *s*_1_ (*x*, *y*) to *S*_0_ (ξ, *η*) and *S*_1_ (ξ, *η*), converting the problem to the frequency domain from the spatial domain. In this process, the image is a discrete source of information and the underlying Fourier transform becomes a discrete Fourier transform.

$$\begin{array}{l} F(u,v)=\underset{x=0}{\overset{M-1}{\sum }}\underset{y=0}{\overset{N-1}{\sum }}f{\left(x,y\right)}^{-2\pi i(\frac{xu}{M}+\frac{yv}{N})} \end{array}$$

$$\begin{array}{l} f(x,y)=\frac{1}{MN}\underset{x=0}{\overset{M-1}{\sum }}\underset{y=0}{\overset{N-1}{\sum }}f{\left(x,y\right)}^{-2\pi i(\frac{xu}{M}+\frac{yv}{N})} \end{array}$$

The above equations describe the conversion from a 2D matrix representation of the image, *f*(*x*, *y*) to the frequency domain *F* and the reverse process. Following that, the ratio of the 2 images is taken in the frequency domain to determine the rotation angle of the target needed to align with the source.

##### ITK Registration Methods

ITK takes an input of 2 images: the source and target. The source is the image to transform to be aligned with the target. The source and target are input into 2 interpolators with a similarity metric process that assesses how closely aligned the source is to the target. With a predefined threshold set, the image iterates through the loop driven by the optimizer algorithms that continues transforming the image until convergence is met. There are 4 main software components of the ITK registration workflow: transformations, the interpolator, the similarity metric, and the optimizer.

#### ITK Transformations

Transformations in the context of image registration and ITK moves points from one space to another—the input to output space.^13^ Medical images and MR scans are in a voxel coordinate space and need to be converted into physical coordinate space before any transformations can occur. ITK has its own C++ classes representing certain important geometric properties of images for optimal transformation. These geometric objects are ITK Point, Vector, and CovariantVector. ITK also requires the Jacobian matrix in order to perform transformations. In the matrix, the elements represent the degree of change a transformation will have on the input space to the output space for each point.

##### Linear Geometric Transforms

These are transformations where a function maps the pixels from one space to another expressed as follows: *T*: *R*^*n*^ → *R*^*m*^. In order for the transform to be linear, it must meet the following criteria:

$$\begin{array}{l} T\left(\vec{u}+\vec{v}\right)=T\vec{u}+T\vec{v} \end{array}$$

$$\begin{array}{l} T\left(c\vec{u}\right)=cT \end{array}$$

All linear transformations are achieved using matrix multiplication and addition, keeping the vector space the same.

##### Affine Transformation

Affine transformation is the simplest and most widely used linear transforms that treats the image as a rigid body. The affine family encompasses all rigid body transforms and contains operations that are uniform and nonuniform scales, rotations, shears, and reflections. It provides 12 degrees of freedom in the 3D space. The mathematical operations applied are straightforward and not computationally intensive. The matrix operation, below, for a rotation in 2D illustrates an affine transformation.^14^

$$\begin{array}{l} \left[\begin{array}{ll} cos(\theta ) & -sin(\theta )\\ sin(\theta ) & cos(\theta ) \end{array}\right]*\left[\begin{array}{l} x\\ y \end{array}\right] \end{array}$$

These affine transforms are composited together to produce the desired alignment, dictated by the metric and optimizer. The crux of the registration difficulty comes with optimizing the transform. An example of a translation of a point is as follows:

$$\begin{array}{l} \left[\begin{array}{l} x^{\prime }\\ y^{\prime } \end{array}\right]=\left[\begin{array}{l} x\\ y \end{array}\right]+\left[\begin{array}{l} t_{x}\\ t_{y} \end{array}\right]=\left[\begin{array}{l} x^{\prime }+t_{x}\\ y^{\prime }+t_{y} \end{array}\right] \end{array}$$

#### ITK Interpolators

The interpolator functions similarly to interpolation in general image processing. Interpolation is the process where one image is remapped onto a new image space through transformations. In order to determine the new image pixels after transformation, interpolation is necessary. Since the advent of image processing and manipulation software such as Adobe PhotoShop, there are some default interpolators used universally for general image manipulation that ITK employs. ITK utilizes the following interpolation algorithms: Nearest Neighbor, linear, b-spline, and windowed sinc interpolation (higher order).^13^

#### ITK Similarity Metrics

The similarity metric is primarily responsible for comparing how closely 2 images are to each other based on a predefined parameter of comparison. This is a crucial process that can significantly affect the resulting registration. A similarity metric can also be used during texture analysis. The metric that is utilized depends on the kind of image data. With unimodal images, it is preferable to use an intensity based metric. In contrast, a multimodal image set is better suited to a mutual information similarity metric. Since ITK v3, the number of similarity metrics has been refactored and reduced. Metrics included in ITK v5 are as follows: mean square, correlation, mutual information, joint histogram/mutual information, demons, and ANTS neighborhood correlation metrics.

##### Means Square

The means square method for assessing similarity between images compares pixel intensities at a given coordinate. This method is pixel intensity driven in the grayscale and is quick to compute. If images *A* and *B* are represented by a matrix, *i* is the pixel index, and *N* is the total number of pixels, the means square metric is calculated as follows^13^:

$$\begin{array}{l} MS(A,\;B)=\frac{1}{N}\underset{i=1}{\overset{N}{\sum }}{\left(A_{i}-B_{i}\right)}^{2} \end{array}$$

A value of 0 indicates that *A* and *B* are the same, with increasing values indicating increasing dissimilarity.

##### Mutual Information

The mutual information method is an area-based method and can be readily applied in assessing the similarities of 2 images being registered. The basis of mutual information comes from the entropy of one random variable to another. Entropy is the measure of randomness of a random variable that is computed using the formula for Shannon entropy^15^:

$$\begin{array}{l} H(A)=-\underset{a}{\sum }p_{A}(a)*log(p_{A}(a)) \end{array}$$

The mutual information in terms of entropy is written in the following 3 equivalent ways:

$$\begin{array}{l} I(A;B)=H(A)+H(B)-H(A,\;B) \end{array}$$ (1)

$$\begin{array}{l} =H(A)-H(A\;|\;B) \end{array}$$ (2)

$$\begin{array}{l} =H(B)-H(B\;|\;A) \end{array}$$ (3)

The mutual information expressions above are analogous to conditional probability. *I* (*A*; *B*) in the second equation states that based on the knowledge of *B*, there is a decrease in the uncertainty of *A*. For MRI images, the random variables are the source and target. To interpret mutual information in the context of equation 2: image *A* at pixel *a* is the uncertainty of *a* minus the uncertainty of pixel intensity given the corresponding pixel intensity at *b* is the mutual information of *a* and *b*.^16^ Achieving the maximum mutual information indicates a successful registration. The uncertainty comparison between source and target demonstrates how the mutual information metric works on multi-modal image sets performing a relative comparison of intensity values putting it closer in line with feature and area based methods versus intensity-based methods.

#### ITK Optimizers

Optimization is the last step in the iterative process of registration. The optimizer’s function consists of a cost function that takes the output value from the similarity metric to calculate and determine the next set of transform parameters to decrease the next metric value. This is an iterative process, of which ITK has many to choose from depending on the transition and metric used.^13^

### Normalization

Normalization is the process by which gray or color values across multiple images are scaled down to a common set of relative gray values. This ensures that variation in acquisition parameters among scanners is accounted for and that similar tissues appear in a common range of values across all images. The classic method for normalization is histogram matching; however, other methods are better suited for MRI images, such as nonparametric and nonuniform intensity normalization.^17,18^

### Skull Stripping Used When Studying the Brain

Skull stripping, or brain extraction, is a computational process that removes extraneous material not critical for analysis such as the skull, fat, and skin.^19,20^ The removal of extraneous information reduces the amount of noise in the system creating a cleaner platform from which features can be segmented and further analyzed. Because the problem is well defined, the process has been refined to where fully automated methods often do a clean job. The skull appears as a bright ring surrounding the brain allowing for an accurate mask to be created for brain extraction. The Brain Extraction Tool (BET) from FSL is an excellent, fully automated process that performs this task with great success.

## Segmentation

**Figure 2.**
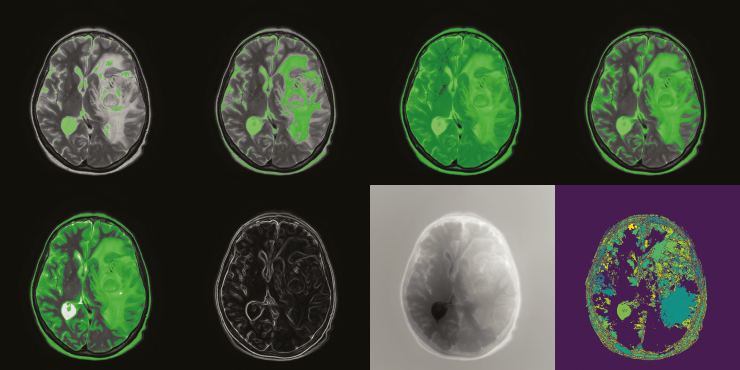
The application of 4 common filters used for segmentation in Insight ToolKit. From left to right and top to bottom, the filters are as follows: simple thresholding, binary thresholding, Otsu’s thresholding, region growing, confidence connected, the gradient magnitude, fast marching, and watershed. It is important to note that none of the parameters have been tuned for any of these filters.

Segmentation occurs after preprocessing and is where an image is divided into disparate, nonoverlapping regions whose texture features share degrees of homogeneity. In patients with brain cancers, the goal would be to delineate the ROI containing tumor, edema, or other distinguishing features. Segmentation of tumors is a very important part of general clinical diagnosis that also forms the basis of imaging studies. In most segmentation challenges, segmentation algorithms are assessed by the accuracy of segmentation of white matter, gray matter, and cerebrospinal fluid. Over the years, segmentation strategies have been developed and are categorized in different ways. There are 3 main types of segmentation that range in their degree of computer-aided automation: manual segmentation, supervised, and unsupervised.^6^ Manual segmentation requires the expertise of a neuroradiologist to draw a perimeter around the area containing the pathology and is completely computer unaided. Supervised segmentation involves input from the user, instructing the algorithm how to perform and what constraints to abide by. The most difficult is unsupervised segmentation method, which requires no user input. Unsupervised segmentation is an area of active research. It is especially problematic with gliomas due to the nature of the disease and surrounding tissue. In some cases, regions can be segmented during the registration process as some alignment functions also recognize distinct regions. A visualization of some of the common segmentation filters applied to an example image can be found in Figure 2.

### Segmentation Methods

#### Region-Growing Algorithms

Region growing is a contextual form of segmentation that accounts for the distance of pixels to the current region at hand. Region growing algorithms are considered classical methods that form the foundation for complex permutations of region growing based methods. The basis of region growing is that a random pixel (seed point) is selected either manually or by the computer and the region around that chosen pixel is compared to its neighbors. Similar pixels are grouped according to some parameter as the region grows out from that seed point. Although this method is conceptually quite simple, it can be overly sensitive. Thus, most software packages that utilize region-growing-type algorithms take into account those shortcomings and have developed some complexity.

##### Connected Threshold

One type of region growing method implemented in ITK is thresholding, specifically connected thresholding. Thresholding turns a grayscale image to a black and white scale by changing each pixel to either black or white depending on a specified gray value cutoff. For example, a simple rule may specify that all pixel values less than constant T will be black and those greater than or equal will be white. The connected threshold method in ITK takes in several parameters as user input: the random coordinates (seed), and upper and lower bounds for the intensities of the region growing algorithm represented as follows: I(X)∈[lower, upper].^13^ As these 3 parameters are required, it is a semiautomatic process. The bounds for the intensities can be determined by observing where the maxima lie on the histogram, calculated either before running through the main region growing algorithm or through observation. Usually, the values for the threshold will lie between 2 maxima. Once the 3 parameters are calculated and input, the process of region growing and thresholding begins by visiting neighboring pixels and determining if they fall under the interval. The process is quick with low computational requirements. Due to the simplicity of the algorithm, it is susceptible to noise and complicated patterns such as inhomogeneities and disconnected regions. This algorithm is ideal for quick prototyping but is limiting.

##### Neighborhood Connected Segmentation

The neighborhood connected method is similar to the connected threshold method. The main differences are that instead of only looking at the next pixel from the current working pixel, the algorithm looks at a neighborhood of pixels and their intensities, like that of a kernel. In this context, a kernel is a fixed square matrix with real number values that iterates over an image from its center point. Depending on the filter, a set of algebraic operations is performed on the current working pixel *I* (*x*, *y*) replacing its value with the new one computed from the kernel.

##### Otsu’s Segmentation

MR images are grayscale with a typical bit depth of 8 (ie, 8-bit images) where each pixel carries 256 (2^8^) gray level values. Otsu’s algorithm is an automated binarization method that attempts to separate the foreground from the background by minimizing the within class variance. The problem is essentially divided into 2 parts: background and foreground. For each part, the weight, mean, and variances are calculated as the algorithm iterates along each threshold value (0–255). Although this algorithm works, it is not the most computationally efficient. The process can run faster by using between class variance and optimizing for the largest value.

##### Confidence Connected

This semi-automatic method utilizes basic statistical features of the image to apply the filter. Here, the user provides a numerical constant and starting seed location. The method calculates the mean intensity and standard deviation of the region and defines an interval based off the constant value provided. This interval given image is represented as follows: I(X)∈[μ−cσ, μ+cσ]. Neighboring pixels that fall in the interval are found and kept record. This process iterates for either a specified number of iterations or until no more pixels fall under the interval. A pitfall of this method is that the region growing is susceptible to incorrect segmentation when the tissue is statistically inhomogeneous. The output is a binary image with a mask, where the segmented region appears in white and the rest in black.

##### Watershed Algorithm

In nature, land topography dictates how water flows and watershed algorithms in segmentation emulate this. By analyzing the topography of the landscape, the problem is redefined using gradient descents.

Gradient descent is an iterative optimization algorithm that attempts to find the local minima of a function and is widely used in machine learning. In this case, the image is represented as a height function whose minimum is sought. There are 2 ways to optimize the function, by either starting from the bottom and finding the maximum or starting from the top and finding the minimum. The ITK framework employs the latter.

#### Level Set Algorithms

The level set family of algorithms originated from the research conducted by Sethian and coworkers, who developed an algorithm that can automatically track curves in any dimension.^21^ The level set methodologies have been applied to other fields, including medical image analysis, and form the basis of a family of segmentation algorithms. The fundamental problem is to accurately model a curve. The straightforward way is to parameterize a curve with a set of explicit equations. This approach, however, is both complex and computationally intensive. Additionally, limitations arise when boundaries intersect, divide, and rejoin over time. To solve this problem, the level set method builds the curve as it propagates in space. The initial level set, where the curve has no change in elevation, is called the zero level set and is represented by φ(x, y)=0. The 2 main ways to describe the curve are through its normal and tangent vectors $\vec{N},\;\vec{T}$ both of which are related to the gradient of *φ*. The other main property of a propagating curve is its velocity *V*. The normal vector is defined by $\vec{N}=-\frac{\nabla \phi }{|\nabla \phi |}$ and the tangent is defined by $\vec{T}=\frac{\nabla \phi }{|\nabla \phi |}$. The normal vector is negative to ensure it points in the inward direction of the curve. The curve’s movement is described in terms of both the explicit curve *C* and implicit curve *φ*. The curve *C* and its movement are described as a function of time with $\frac{dC}{dt}=V$ and is related to the implicit definition by $\frac{d\phi }{dt}=V|\nabla \phi |$. This forms the basis of the level set methodology.

ITK represents the level set function as a higher dimensional function from the beginning as  Ψ (X, t) where the zero level set is  Γ (X,t)={ Ψ (X,t)=0}.^14^ Here, *X* refers to the *n*-dimensional surface and *t* the time step. Internally in ITK, the level set works via the following general partial differential equation:

$$\begin{array}{l} \frac{d\;\Psi \;}{dt}=-\alpha A(x)*\nabla \;\Psi \;-\beta P(x)\left|\nabla \;\Psi \;\right|+\gamma Z(x)\kappa |\nabla \;\Psi \;| \end{array}$$

In the equation, *α*, *β*, *γ* are constants that serve as weights to influence the advection, propagation, and spatial modifier for the curvature, respectively.

##### Fast Marching Segmentation

The fast marching method is a level set that can quickly resolve shapes when the problem is fairly simple. In fast marching, the problem is framed around movement of the curve starting from the zero level set ϕ(x,y)=0 and the propagating speed of the curve *F*(*x*, *y*) > 0.^22^ Fast marching functions by aiming to solve the Eikonal partial differential equation, an equation used to model many physical phenomena. The solution to this equation is a set of points, which is the curve and verified to be accepted. In ITK, this starting set of points is user provided as a seed point for the algorithm to start its curve propagation. Because the level set family of algorithms is able to merge with other growing curves, it is preferential to even use multiple seed points for efficient computation.

##### Shape Detection

Shape detection was pioneered by Malladi and Sethian and forgoes the parameterized, geometric Lagrangian approach taken by earlier “snake” methods for level sets.^21^ The ITK shape detection filter implements Malladi’s principles by requiring 2 objects of input: the initial ITK image as a level set and its edge potential image, produced via sigmoid filter, which is used to help determine the speed of front propagation. Before the original image goes through the shape detection module, it is first preprocessed with a Gaussian filter, followed by the sigmoid filter to create its complementary edge potential image. Briefly, the process is:

1. Read image with ITK
2. Smooth with anisotropic filter
3. Smooth again with Gaussian filter
4. Produce edge potential image with Sigmoid filter
5. Use seeds and distance parameters to create a level set from the ITK image
6. Pass level set and edge potential into shape detection module and post process with a binary filter to reveal the segmented image

##### Geodesic Active Contour

The Geodesic active contour method, proposed by Caselles et al., sought to solve the limitations of the classical “snake”-based method of curve tracking that fails when topological changes are presented.^18^ This is achieved by starting from the classical snake’s energy-based representation of the curve, called *E* (*C*) and expressed as follows:

$$\begin{array}{l} E(C)=\alpha \underset{0}{\overset{1}{\int }}|C^{\prime }\left(q\right){|}^{2}dq-\lambda \;\underset{0}{\overset{1}{\int }}|\nabla I(C(q))|dq \end{array}$$

Here, *α* and *λ* are constants greater than 0, the first integral represents the contour’s smoothness, and the second the attraction of the contour to an arbitrary object in the image *I*.^23^ Maupertuis’ and Fermat’s Principles are combined with Sethian’s level set to derive the implicit parameterization of curves via geodesics.

In ITK, this underlying theory is abstracted to a workflow similar to that of the shape detection. For the ITK geodesic pipeline, the parameters that can be changed affect the propagation, curvature, and advection of the curves that are drawn from the source image. The pipeline parameters are: seed coordinate, distance, σ for the sigmoid filter, *α* and *β* constants, and a propagation scaling value.

##### Canny Edge

The last commonly used segmentation method found in ITK, as well as SciKit and OpenCV, is Canny edge detection, which works by calculating the gradient of the image and using the resulting matrices to “find” the edge. A Gaussian filter is commonly applied first to the image to remove noise and smooth edges. In the ITK workflow, the 2 parameters that can be modified are the variance for the Gaussian filter, and a threshold value for the binary thresholding at the very end.

### Atlas-Based Segmentation with a Focus on Brain Imaging

Unlike previous techniques, atlas-based segmentation is not a de novo technique. An atlas is a template that outlines and defines the main anatomical structures and their coordinates, typically on the 3 anatomical planes (axial, sagittal, coronal). For brain imaging, an atlas of a healthy human brain is used to perform and aid in segmenting features. Several standards and types of atlases exist including the Talairach Atlas and Allen Brain Atlas. The 2 main types of atlases are topological (deterministic) and probabilistic.^24^ The Talairach atlas is topological and attempts to map out a healthy male and female brain volumetrically using a combination of imaging modalities such as CT and MR. It is often sourced from only one sample. Probabilistic atlases, in contrast, are created from multiple subjects in order to probabilistically determine the chances of a certain feature appearing in a certain region of the brain. It is akin to the creation of a probability distribution for a random variable of brain atlases. These atlases address the shortcomings of the Talairach atlas by establishing a probabilistic map of brain tissue features often produced from a large sample of subjects. A major source of data for probabilistic atlases comes from the UCLA Brain Mapping Center, part of the International Consortium for Brain Mapping. Using these atlases, it is possible to segment features from new scans. The first step is typically to undergo preprocessing steps that involve skull stripping and image registration to the atlas. Once completed, there are 3 main atlas-based segmentation strategies that can be utilized: label propagation, multiatlas propagation, and probabilistic atlas segmentation.

The simplest method is label propagation, which assumes that once the image is registered to the atlas, many of the major anatomical structures are approximately in the same voxels. The general framework of label propagation algorithms attempts to map the labels from the atlas onto the image of interest. These mappings are almost like a continuation of registration, as the mathematical techniques often used are ones such as affine transformations and principle axes. More complex methods can also be used, such as the level set-based approaches. Label propagation is limited as it simply outlines major contours and cannot identify new features.

Multiatlas propagation is the application of label propagation across multiple atlases. The biggest challenge with this approach is choosing how to aggregate and register the labels across multiple atlases. One common method is to use a weighting function for each atlas to classify voxels and has seen favorable accuracy. For general probabilistic segmentation, the approach is Bayesian and expressed as p(l(x) | c)∗p(c); the conditional probability of the pixel intensity given a class *c* (label of a feature) and the class prior *p*(*c*).^24^ The probabilistic strategy tends to work best when segmenting new features, such as tumors.

#### Segmentation and Brain Tumors

The preprocessing steps prior to segmentation are necessary to increase the probability and quality of accurate segmentation. In the clinical setting, a licensed radiologist parses through a patient’s data, identifies key features through segmentation, and reports their findings—an arduous process that takes years of experience and time. Computationally driven segmentation of brain tumors is necessary to reduce this overhead while procuring the same quality of information for data driven studies. However, gliomas, the most common type of malignant brain tumor in adults, can manifest in any region of the brain and are much harder to detect when they are lower grade. Fortunately, the availability of neural network frameworks has given researchers a new tool to address the segmentation challenge.

## Feature Extraction

Once the ROI have been accurately segmented and classified, the next step is to find meaning from the newly sorted information through feature extraction. The major features that are used are first order, gray level co-occurrence, structural, and transform features. Each of these provides information about an image or image series.

### First-Order Features

First-order statistical features are those that are directly computed from the gray value intensities in the image. These are computationally simple and form the basis of second and higher order features. Table 1 summarizes the most used first-order features for texture analysis where *m*, *n*, and *f* refer to the length, width, and the image, respectively.^13,17,25^

**Table 1.** A Summary of Common First-Order Statistical Features and Their Significance in regards to a Grayscale Image

Feature	Formula	Significance
Mean (M)	$M=\frac{1}{m*n}\underset{x=0}{\overset{m-1}{\sum }}\underset{y=0}{\overset{n-1}{\sum }}f(x,y)$	The average gray-level value taken across all pixels.
Standard deviation (SD)	$SD=\sqrt{\frac{1}{m*n}\underset{x=0}{\overset{m-1}{\sum }}\underset{y=0}{\overset{n-1}{\sum }}{\left(f(x,y)-mean\right)}^{2}}$	Second central moment that indicates inhomogeneity. Higher the SD, higher the contrast.
Entropy (*E*)	$E=\underset{x=0}{\overset{m-1}{\sum }}\underset{y=0}{\overset{n-1}{\sum }}(f(x,y)\log_{2}f(x,y))$	Indicates the degree of randomness in the image.
Skewness (*S*_k_)	$S_{k}=\frac{1}{m*n}\times \frac{\overset{}{\sum }{\left(f(x,y)-M\right)}^{3}}{SD^{3}}$	Indicates the degree of symmetry of gray values centered about the mean.
Kurtosis (*K*)	$K=\frac{1}{m*n}\times \frac{\overset{}{\sum }{\left(f(x,y)-M\right)}^{4}}{SD^{4}}$	Describes the image’s distribution of gray values relative to the mean vs the tails.
Energy (En)	$En=\sqrt{\underset{x=0}{\overset{m-1}{\sum }}\underset{y=0}{\overset{n-1}{\sum }}(f^{2}(x,y))}$	Describes the degree of pixel value pair repetitions in the image.
Contrast (*C*_on_)	$C_{on}=\underset{x=0}{\overset{m-1}{\sum }}\underset{y=0}{\overset{n-1}{\sum }}({\left(x-y\right)}^{2}f(x,y))$	Describes the overall measure of intensity of pixels compared with its neighbors.
Inverse difference moment (IDM)	$IDM=\underset{x=0}{\overset{m-1}{\sum }}\underset{y=0}{\overset{n-1}{\sum }}\frac{1}{1+{\left(x-y\right)}^{2}}*f(x,y)$	Quantifies the homogeneity of the image.
Directional moment (DM)	$DM=\underset{x=0}{\overset{m-1}{\sum }}\underset{y=0}{\overset{n-1}{\sum }}f(x,y)*|x-y|$	Measures the alignment of the image.
Correlation (*C*_orr_)	$C_{orr}=\frac{\overset{m-1}{\underset{x=0}{\sum }}\overset{n-1}{\underset{y=0}{\sum }}((x,y)f(x,y))-M_{x}M_{y}}{\sigma_{x}\sigma_{y}}$	Measures the degree of linearity in an image (shows linear structure like striations).
Coarseness (*C*_ness_)	$C_{ness}=\frac{1}{2^{m+n}}\underset{x=0}{\overset{m-1}{\sum }}\underset{y=0}{\overset{n-1}{\sum }}f(x,y)$	Quantifies the roughness of the texture in the image.

### Gray-Level Co-occurrence Matrices

Gray-level co-occurrence matrices (GLCM), developed by Haralick et al., show the occurrence of gray levels per pixel relative to other pixels.^26^ These matrices can show the run length of the gray level values in 4 directions *θ*: 0°, 45°, 90°, 135°. Among the most common GLCM are the gray level run length matrix (GLRLM), gray-level size zone matrix (GLSZM), neighborhood gray tone difference matrix, and gray-level dependence matrix.

A GLCM has square dimensions of length equal to the number of gray values. The matrix is computed by counting the frequency of gray value *i* to gray value *j* per a determined spatial relationship. The most common spatial relationship used is adjacency to the current pixel. A GLRLM maps out how many continuous gray-level values exist in the image along a defined angle *θ*. For example, if an image has 8 gray-level values and is of dimension 10 × 10 pixels, the resulting GLRLM has dimensions 8 × 10. The rows represent each gray-level value and the columns represent the length of contiguous pixels of that gray-level value. The number of unique occurrences is counted. A GLSZM is similar to the GLRLM in that continuous gray-level values are counted, with the added condition it counts every single connected instance not restricted by an angle *θ*. This results in only one matrix. With the results taken from the first and GLCM statistics, models to characterize images or an image series are built (3D).

### Structural Features

Structural features, or morphological features, describe the shapes of a ROI. Common 3D structural features are volume and shape metrics.^27^ Volumetric features include volumes of contrast-enhanced tumor, peritumoral edema, necrosis, and nonenhancing tumor. Ratios of each of these regions can be taken to compute a comparative metric. Shape features include the bounding ellipsoid volume ratio, which is the ratio of the tumor’s volume to the volume of the smallest ellipsoid that bounds the tumor. The orientation of the ellipsoid highlights the spatial position of the tumor. Metrics of sphericity, measuring roundness, compare the ratio of the surface area of the tumor to the surface area of a sphere of equivalent volume. In addition to 3D features, 2D shape features are computed on a per slice basis and include a tumor’s centroid, mean radial distance, radial distance standard deviation, mass circularity, entropy of radial distance, area ratio, zero crossing count, and mass boundary roughness.

### Transform Features

The other approach to extracting features is by decomposing the image into a frequency domain allowing for a spectral analysis approach. Common transform methods are wavelet transforms, Fourier, and discrete cosine.

### Statistical Tests

Once the features have been extracted, perhaps the largest hurdle is result interpretation and statistical testing. When testing for normality, tests such as the *t*-test, ANOVA, the Kruskal–Wallis, and Mann–Whitney are used. When multiple groups with multiple features exist, the Tukey honest significant difference test or Benjamin–Hochberg tests are utilized. For a logistic regression type analysis, the standard Cox regression model and receiver operating characteristic analysis are often used.^28^

### Biomarker Recording

All of the features discussed quickly create a massive matrix of data, making it difficult to properly maintain records of each biological feature, or biomarker. To address this data management problem, the image standardization biomarker initiative (IBSI) was founded to devise a set of rules to standardize the extraction and naming of imaging biomarkers, enhance reproducibility, suggest workflows, and establish biomarker reporting guidelines.^29^

**Figure 3.**
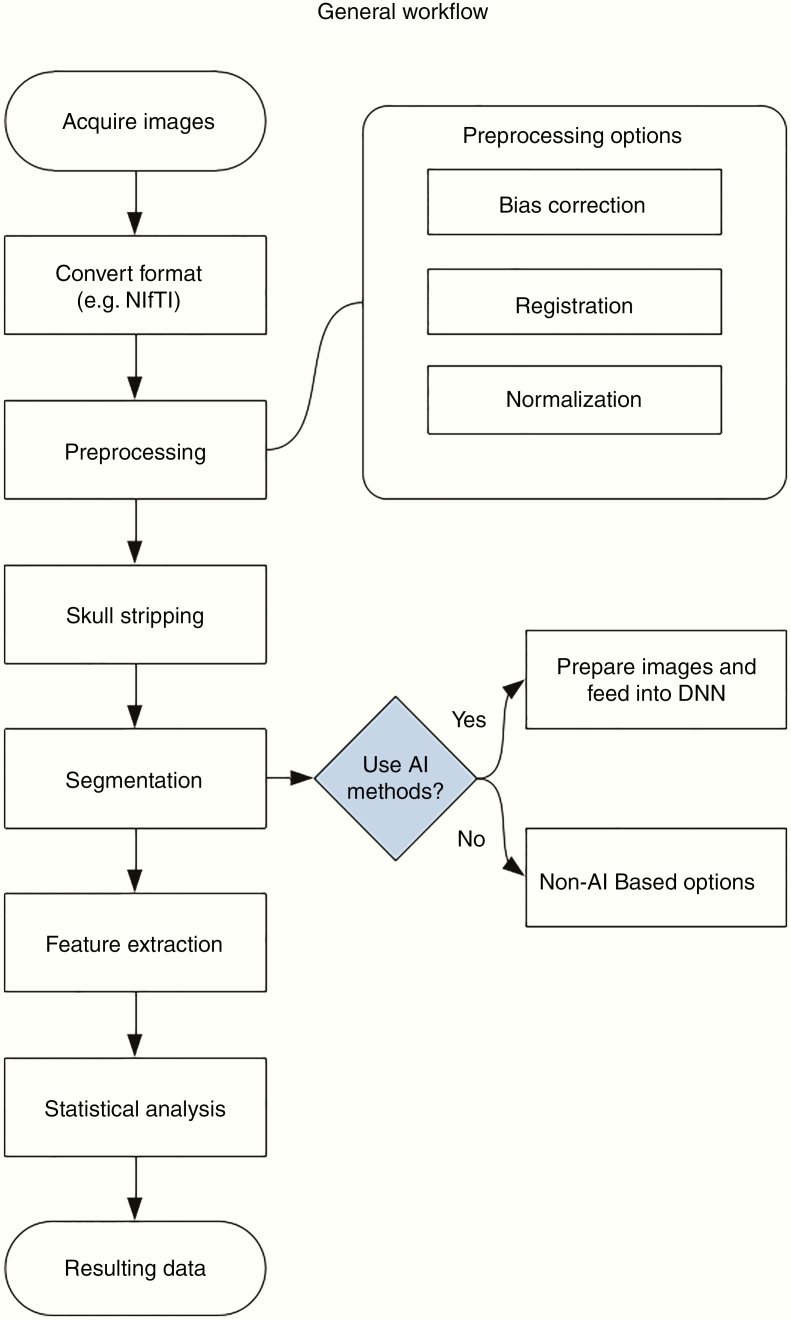
A flowchart of a general MR image analytics workflow and a potential use of AI-based methods in the segmentation process block.

The IBSI has proposed a general scheme for biomedical image processing workflows. As the field is dynamic, this scheme is not permanent but rather a guideline for investigators. This review has been structured in a way that follows IBSI’s scheme: data acquisition, preprocessing, segmentation, image interpolation (optional), feature extraction, and feature data. A high-level visualization of this workflow can be found in the flowchart in Figure 3. Interpolation is optional in cases where patients do not have the same number of slices. This often occurs in multi-institutional studies. Interpolating missing images for parity is sometimes necessary, depending on the type of analysis to be performed.

The main quantitative image features that the IBSI outlines are morphology, local intensity, intensity-based statistics, intensity histogram, intensity volume histogram, gray level co-occurrence, run length, size zone, distance zone matrix, neighborhood gray tone difference, and neighborhood gray-level dependence matrix. For each of the features in these groups, IBSI assigns a standard code. For example, the mean intensity statistical feature is assigned the ID of Q4LE. To further remove ambiguity and avoid the misuse of terminology when discussing certain radiomics terms the nomenclature is defined. The guidelines set forth by IBSI are thorough and outline a typical image processing workflow for investigators.

### Approaches Using AI

AI is a relatively new field that emerged in the mid-20th century. AI can be generally defined as the study of rational agents, their composition, and construction that encompasses both machine learning and deep learning approaches.^30^ Minsky and Papert formulated the early theory of perceptron which was a generalizable model establishing the foundation of neural networks that comprises the basis of many modern deep learning models today. Neural networks are made up of nodes (neurons), an activation *a*, and a set of parameters  Θ ={W,B}, which are weights and biases, respectively. The activation is simply a linear combination of the input *x* to the parameters multiplied by a transfer function *σ* which is expressed as $a=\sigma (w^{T}x+b)$.^25^ Common transfer functions are the sigmoid and hyperbolic tangent functions. These inputs **x** undergo numerous transformations that in turn form the hidden layers of a deep neural network (DNN). One of the most widely used DNN, especially in MRI imaging, is the convolutional neural network (CNN).^31^ Its use can be observed in all parts of the workflow.

Among these DNN, some of the most widely used network architectures are ResNet, generative adversarial neural networks (GANs), and U-nets. The last of which is particularly important as the authors who formulated the U-net architecture did so with a focus of applying it to segment medical data.^32^ The foundational blocks that comprise a CNN are its convolutional and pooling layers. A convolutional layer, which takes a layer of neurons as input, applies a filter to that layer of neurons. The raw input image is the initial layer set followed by filters to produce a feature map of the original data. This is then passed further through the network. The feature map is what the network deems as a unique feature. Often, the convolutional layers and its filters will produce a vast number of features in its map. When this occurs, a pooling layer is added, condensing the feature map to reduce its size. The third important block is batch normalization. As the name suggest, batch normalization normalizes data and consequently accelerates the learning process in the CNN. This is achieved through normalization of new inputs before each layer. In constructing these CNNs, network architects have increased freedom to choose the location and number of the convolutions, in addition to other features. This ability allows for the generation of unique networks that can be used to accomplish individual imaging goals. In contrast to traditional methods where registration and segmentation can be answered by framing the problem in different ways, deep learning accomplishes this through the construction of novel networks, training it with a large data set, and assessing the results.^33^

Deep learning approaches also start and end differently compared with traditional methods. Deep learning algorithms require a training set of already preprocessed, normalized images that are cropped to the same dimensions. This is crucial as the quality of the input dictates output quality. Although the aforementioned methods in ITK function can successfully segment disparate regions, they do require manual tweaking, which becomes cumbersome with large data sets. With deep learning, the CNN performs the tweaking automatically while iterating through the convolutions. Applications of deep learning have been used in all aspects of MRI image data including image registration, segmentation, and feature extraction and classification.^18^ Besides the application of CNNs to address the traditional problems, they can be used unconventionally to generate artificial data via GANs. This has led to research in ways GANs can be used to denoise data and find artifacts.^31^ Recent developments in this area have been the use of GANs to super-sample low resolution MRI images to create resulting data that has effectively higher spatial resolution than the source while maintaining source structural integrity.^34^

#### Neural Networks and Brain Tumors

When applied to general imaging analytics, neural networks have had some success when compared with prior methods, which was related to previous over tumor segmentation in MRI images.^35^ Segmentation of brain tumor features is challenging due to the wide variability at present and progression of disease, making the accuracy of CNNs more attractive for use in this complex disease. Unlike tabulated information, 3D MRI scans contain vast amounts of information. When training a model using imaging data, a CNN can often times create millions of parameters as it attempts to find and classify features.^35^ Typically, MRI images can be fed into a model by dividing each slice into patches or by supplying the whole slice image. Zhao et al. employed a fully convolutional neural network that was found to be more efficient by reading the full slice.^35^ As these novel approaches are more commonly applied to brain tumors, it is expected that novel discoveries for patient translational will be utilized.

## Concluding Remarks

MRI imaging analysis advanced significantly since the advent of computer vision and computer graphics. Many advances were made in parallel and led to the creation of key tools such as ITK and FSL. Both are widely used among researchers with continued refinement. AI is being applied to many areas, including MRI imaging analysis, which is now moving at an accelerated pace as new deep learning-based research is conducted. This application of AI will undoubtedly open new areas of research and investigation, particularly for challenging diseases such as brain tumors.

## Funding

This work was supported through developmental funds from CWRU School of Medicine and University Hospitals Research Division.

*Conflict of interest statement*. None of the authors have any conflicts of interest to disclose.

## Authorship Statement.

All authors participated in the manuscript draft and revision.
